# PROX1 promotes breast cancer invasion and metastasis through WNT/β-catenin pathway via interacting with hnRNPK

**DOI:** 10.7150/ijbs.68960

**Published:** 2022-02-28

**Authors:** Lizhe Zhu, Qi Tian, Huan Gao, Kaijie Wu, Bo Wang, Guanqun Ge, Siyuan Jiang, Ke Wang, Can Zhou, Jianjun He, Peijun Liu, Yu Ren, Bin Wang

**Affiliations:** 1 Department of breast surgery, the First Affiliated Hospital of Xi'an Jiaotong University, Xi'an, China.; 2 Department of medical oncology, the First Affiliated Hospital of Xi'an Jiaotong University, Xi'an, China.; 3 Department of Urology, the First Affiliated Hospital of Xi'an Jiaotong University, Xi'an, China.; 4 Center for Translational Medicine, the First Affiliated Hospital of Xi'an Jiaotong University, Xi'an, China.

**Keywords:** PROX1, breast cancer, EMT, WNT signaling, hnRNPK

## Abstract

**Background:** The progressive, multifactorial and multistep dynamic process of metastasis is the primary cause of breast cancer (BC) lethality. PROX1 (Prospero-related homeobox 1), as a type of transcription factor that plays a key role in the formation of lymphatic vessels in animal embryonic development, has been proven to promote or suppress cancer in a variety of malignant tumors. However, molecular mechanisms behind PROX1 induced breast cancer metastases remain elusive.

**Methods:** Changes of PROX1 expression and clinical significance of PROX1 in BC were evaluated by BC tissue, as well as public database. The functional role of PROX1 in metastases BC was analyzed by transwell assay *in vitro*, and by lung metastases model of nude mice *in vivo* via lentivirus mediated knockdown assays. Mechanism studies were performed by public database screening, western blot and PCR assay, immunoprecipitation, immunofluorescence staining and luciferase promoter assays.

**Results:** In this study, we found that PROX1 was upregulated in breast cancer tissues; increased PROX1 expression in breast cancer was associated with tumor size, lymph node metastasis, ER and PR status. Meanwhile, PROX1 can promote breast cancer invasion and metastasis *in vitro* and *in vivo*. Furthermore, PROX1 can interact with hnRNPK to activate WNT/β-catenin signaling in breast cancer cells. Moreover, the interaction of PROX1 and hnRNPK inhibits the ubiquitination of hnRNPK, and subsequently activates WNT pathway to promote the invasion and metastasis of breast cancer.

**Conclusions:** In conclusion, our findings indicated PROX1 contributes to breast cancer EMT and metastasis and serves as a candidate diagnostic biomarker and promising therapeutic target for breast cancer.

## Introduction

The latest global cancer statistics in 2020 show that female breast cancer has surpassed lung cancer and ranked first in the global cancer incidence 1. 3%-10% of newly breast cancer patients have distant metastases at the time of diagnosis, and 30%-40% of early-stage patients will develop into advanced breast cancer 2. Therefore, elucidation the mechanisms of metastasis can provide novel therapeutic strategies for patients with metastatic breast cancer.

Since tumor metastasis is the main reason for the high mortality of breast cancer and it is a complicated and dynamic process. Epithelial cell-to-mesenchymal transition (EMT) enables epithelial cancer cells acquire a mesenchymal phenotype which facilitates metastasis. EMT is regulated by a complex network. Important transcription factors include β-catenin, ZEB1, ZEB2, SLUG, SNAIL, and TWIST 3. Among those, Miranda-Carboni's team demonstrated that more than 50% of breast cancer patients have high expression of β-catenin in the tumor cell nucleus, and this proportion is even more than 80% in triple-negative breast cancer (TNBC) 5. In recent years, studies have found that, as an important transcription factor that promotes EMT, β-catenin can abnormally activate, aggregate and combine with TCF/LEF to enter the nucleus and activate the transcription of downstream target genes 6. Classic EMT transcription factors ZEB1/ZEB2 are all regulated by β-catenin. Normal breast tissue β-catenin is only expressed in the cell membrane, while breast cancer tissue β-catenin is expressed simultaneously in the cell membrane, cytoplasm and nucleus. Abnormal expression of β-catenin is related to lymph node metastasis, histological grade, clinical stage, Ki67 status and prognosis 7. Therefore, β-catenin plays an important role in the occurrence, development, and metastasis of breast cancer.

PROX1 (Prospero-related homeobox 1) gene is a type of transcription factor that plays an important role in the formation of lymphatic vessels in animal embryo development. It was confirmed in knockout mice that when PROX1 is lacking, mice cannot normally differentiate into lymphatic endothelial cells. PROX1 maintains its own stability by binding to hormone receptors in the body and plays a role in the early stage of lymphatic endothelial formation 8. In fact, the high expression of PROX1 can promote the formation of lymphatic vessels, which has important pathological significance for lymph node metastasis in cancer. According to different literature reports, depending on the type of cancer, PROX1 not only work as tumor suppressor but also exhibit oncogenic activity. Recently, more and more studies have found that the abnormally high expression of PROX1 is related to many systemic malignant diseases such as neuroblastoma, colon cancer, kidney cancer, gastric cancer, esophageal squamous cell carcinoma and hepatocellular carcinoma. A review of the literature shows that in patients with neurocytoma, the higher the expression of PROX1 in nucleus, the higher the ratio of undifferentiated cells/poorly differentiated cells and the probability of lymphatic metastasis, and the worse the prognosis 9. In colon cancer, PROX1 inhibits the expression of E-cadherin at the transcriptional level by inhibiting microRNA-9-2, thereby promoting EMT and invasion and metastasis 10. Yokobori et al. found that in esophageal cancer, the expression of PROX1 in the nucleus is positively correlated with the expression of HIF1α protein, and negatively correlated with the level of E-cadherin 11. In liver cancer, PROX1 can increase the stability of the transcription factor hypoxia-inducible factor 1α (HIF1α) by recruiting histone deacetylase (HDAC1), leading to EMT of liver cancer cells 12. In renal clear cell (RCC) carcinoma, although the expression of PROX1 is lower than that of the surrounding normal tissues, the relatively high expression of PROX1 in cancer tissues is significantly related to poor prognosis. PROX1 reduces E-cadherin and promotes the expression of vimentin, enhance the proliferation and migration ability of RCC 13. However, the effect of PROX1 on EMT and its influence on the invasion and metastasis of breast cancer remains unclear.

Therefore, this study predominantly focuses on exploring the role of PROX1 in breast cancer *in vitro* and *in vivo*. We reported that PROX1 promote metastasis and invasion of breast cancer through the WNT pathway by interacting with hnRNPK which can facilitate β-catenin nuclear translocation. Moreover, PROX1 intervenes ubiquitination of hnRNPK in breast cancer cells. This study takes the new evidence to support PROX1 as an oncogene in breast cancer.

## Materials and Methods

### Patients and datasets

A total of 1035 breast cancer RNA expression data were downloaded from the TCGA cohort (http://portal.gdc.cancer.gov). In addition, three independent cohorts with breast cancer RNA expression data were downloaded from Gene Expression Omnibus (GEO) database (http://www.ncbi.nlm.nih.gov/geo) including GSE1379, GSE6434 and GSE27447. All the data from TCGA and GEO were public and our study followed the TCGA and GEO data access policies and publication guidelines. Also, the alteration of PROX1 was analyzed online by cBioPortal (http://www.cbioportal.org/). The 74 pairs of breast cancer tissues and adjacent non-cancerous tissue patients were from the First Affiliated Hospital of Xi'an Jiaotong University (Shaanxi, China). All the patients had signed the informed consent before surgery, and did not suffer from other malignancy or received radiotherapy or chemotherapy. Tumor tissue and adjacent normal tissue were confirmed by a pathologist and frozen with liquid nitrogen to prevent protein degradation. The human breast cancer tissues used in this study have received consent from the Ethics Committee of the First Affiliated Hospital of Xi'an Jiaotong University and conducted in conformity to the Declaration of Helsinki.

### Cell culture and reagents

MCF-7 was maintained in DMEM High Glucose with 10% fetal bovine serum (FBS) and 1% penicillin and streptomycin. T-47D was cultured in RPMI 1640 medium with 10% FBS and 1% penicillin and streptomycin. MDA-MB-231 and MDA-MB-468 were grown in L15 medium with 10% FBS and 1% penicillin and streptomycin. SUM159 was cultured in DMEM/F12 medium with 10% FBS and 1% penicillin and streptomycin. BT549 was cultured in RPMI 1640 medium with 10% fetal bovine serum (FBS), 1 μg/ml insulin and 1% penicillin and streptomycin. Medium, FBS and Penicillin-Streptomycin were purchased from Gibco (Thermo Fisher Scientific, Inc., Waltham, MA, USA). All the cells were maintained at 37 °C with 5% CO_2_. Old cell culture medium was replaced with fresh medium every other day. All the antibodies used in this study can be found in Supplementary Table 1. XAV939 and SKL2001 were purchased from Selleck.

### Immunohistochemistry

The paraffin embedded tissue sections were first roasted in 60 °C for 6h and then deparaffinized in xylene, later dehydrated in gradient concentration ethanol. The antigen repair of tissue slides was performed in sodium citrate buffer (PH = 9.0) in microwave oven for 3 min in 100 W and 12 min in 50 W. After natural cooling of sections, 3% hydrogen peroxide was used to deactivate endogenous peroxidase for approximately 10 minutes. Subsequently, the slides needed to be cleaned three times by phosphate buffered saline (PBS) and blocking with 5% bovine serum albumin (BSA) at room temperature for 30 minutes. And then, incubation with primary antibodies in a certain concentration overnight at 4 °C. On the second day, the slides were cleaned by PBS for three times (5 minutes each time) and then incubated in homologous secondary antibody at room temperature for 1h. After 3 times PBS washing again, diaminobenzidine (DAB) staining, hematoxylin staining, dehydration in gradient ethanol and transparency in xylene were followed to handle the slides.

The scoring method was as follows: 10 random fields of each tissue section were selected for semi-quantitative scoring, and the scoring method was as follows: 1) Positive cell rate score 0 for <10% positive cells, 1 for 10%~25% positive cells, 2 for 25%~50% positive cells, 3 for 50%~75% positive cells and 4 for >75% positive cells; 2) Dyeing strength score 1 for light yellow, 2 for brown yellow and 3 for tan; 3) The total score was the product of the positive cell rate score and staining intensity score: 0 as negative; 1-4 as weak positive; 4-12 as strong positive.

### Plasmid transfection, RNA interference and lentiviral infection

Human PROX1 small hairpin RNA (shRNA) and OE-PROX1 lentivirus were obtained from GeneChem (Shanghai, China). MCF-7 cells were infected with lentivirus and taken MOI = 20 as the standard. MDA-MB-231 and SUM-159 cells were infected with lentivirus and taken MOI = 10 as the standard. All cells were infected with lentivirus for sh-NC expression (sh-NC) or control lentivirus (Con) as negative control. All cells were infected using transfection reagents HiTransG P (GeneChem) and treated with medium containing puromycin (Cayman Chemical, Ann Arbor, USA) for three weeks to generated stable PROX1 knockdown or overexpressed cells. In addition, the hnRNPK siRNA molecules were obtained from Gene Pharma, Shanghai, China. The siRNA was transfected into cells using Lipofectamine 2000 (Invitrogen, Carlsbad, CA). All sequences were listed in Supplementary Table 2.

### RNA isolation and real-time qRT-PCR

Total RNA from cell lines was extracted using the RNeasy mini kit (Qiagen, Valencia, CA) according to the manufacturer's protocol. Concentration and purity of all RNA samples were determined at an absorbance ratio of 260/280 nm. And then, a total of 1 µg RNA was reverse-transcribed using iScriptTM cDNA Synthesis kit from Bio-Rad (Hercules, CA). Real-time PCR analysis was conducted with the SYBR Green qPCR Supermix kit (Invitrogen, Carlsbad, CA) and carried out in the iCycler thermal cycler. The relative level of mRNA expression of a gene was determined by normalizing with GAPDH. The primers used for real-time qRT-PCR were listed in Supplementary Table 2 and purchased from Sangon Biotech.

### Western blotting

Whole cell lysates were prepared by RIPA lysis buffer, containing phosphatase and a protease inhibitor (Roche, NJ, USA), by using an ultrasonic crusher (SONICS and MATERIALS INC. USA). Then, the proteins were kept on ice for 20 min and transferred to a centrifuge tube for centrifugation at 4 °C, 20 min. The proteins from the upper-part of the supernatant were collected and detected using a BCA Protein Assay Kit (Pierce; Thermo Fisher Scientific, Inc.). Then, 30 µg of protein was separated on 10% SDS-PAGE gels and transferred to polyvinylidene fluoride (PVDF) membranes (Merck Millipore, Billerica, MA, USA). The membranes were blocked with 5% nonfat milk in TBST to prevent nonspecific binding and subsequently incubated with primary antibody overnight at 4 °C. All samples were incubated with anti-horseradish peroxidase-linked IgG secondary antibody (cat no. 7074; Cell Signaling Technology, Inc.; dilution, 1:2,000) at room temperature for 2 h and detected via chemiluminescence detection system (version 3.0; Bio-Rad Laboratories, Inc., Hercules, CA, USA). Lamin A/C and GAPDH were acted as control. In addition, NE-PER Nuclear and Cytoplasmic Extraction Reagents (Thermo Fisher Scientific Inc. USA) was used for cell fractionation assays.

### Migration and invasion assay

The transwell chambers for the invasion assays were coated with matrigel (1:7 dilution with serum-free medium). 200,000 cells in 200 μl serum-free medium were seeded in the upper chamber (8-μm pore non-coated polycarbonate transwell inserts), and 700 μl 10% FBS-containing medium was added to the lower chamber. The plates were placed in 5% CO2 at 37 °C for 24-72 hours. Then the cells were fixed in 4% PFA (paraformaldehyde) for 10 mins and followed by 5% crystal violet staining for 15 mins. After gently removing the cells on the upside of the top chamber, the migrated or invaded cells were subsequently photographed and counted.

### Immunofluorescence

Cells were fixed with 4% paraformaldehyde, permeabilized with 0.2% Triton X-100, and blocked with 5% BSA. Later, cells were incubated overnight with specific primary antibody, followed by fluorescent-labeled secondary antibody (Proteintech). DAPI was then used for DNA staining. Image capture was performed by a Leica inverted fluorescence microscope and confocal laser scanning microscope (Leica TCS SP5).

### Luciferase reporter assay

Reporter gene transfection and luciferase activity assay were performed as follow: cells in 24 well plates were co-transfected with TOPflash or FOPflash plasmids (Addgene) and Renilla TK-luciferase vector (Promega) as a control using Lipofectamine 2000 (Invitrogen). The luciferase activity was measured by the dual-luciferase assay system (Promega) according to the protocols provided by manufacturers. The firefly luciferase activity was normalized to the internal Renilla luciferase activity, and the β-catenin-driven transcription was measured as TOP/FOP ratio.

### Co-immunoprecipitation and mass spectrometry

Total protein was extracted by NP-40 lysis buffer (20 mM Tris-HCl, 150 mM NaCl, 0.5% NP-40, 20% glycerol) containing phosphatase inhibitors and protease inhibitor (Roche, NJ, USA) and then centrifuged at 12,000 rpm 4 °C for 20 min. Cell lysates were incubated with magnetic beads to prepare bead-antibody-antigen complexes using 4 μg antibody and 50 μl protein A/G magnetic beads (Thermo Fisher, 10007D). After washing with elution buffer, the obtained binding protein complexes were boiled and then tested by Western blotting.

For IP MASS analysis of potential PROX1 binding proteins, eluted Co-IP samples (IP PROX1 in MDA-MB-231 and IP PROX1 in PROX1 overexpressed MCF-7 cells) were resolved on 10% SDS-PAGE and stained with colloidal coomassie. Gel samples lanes were cut into defined pieces, de-stained, and trypsinized. The resulting peptide solutions were extracted, subjected to mass spectrometry (Orbitrap EliteLC-MS/MS, ThermoFisher Scientific, USA), and analyzed using the PEAKS Studio software (Bioinformatics Solutions, Inc., Waterloo, Ontario, Canada) in the Proteomics Platform of Core Facility of Basic Medical Sciences, Shanghai Jiao Tong University School of Medicine (SJTU-SM).

### Ubiquitination assay

Plasmid expressing ubiquitin-HA was infected to cells transiently by Lipofectamine 2000. An additional 8 h MG132 (5 μmol/L, Sigma) treating for cells was necessary after 24h. NP-40 lysis buffer was used to treat cells. Co-IP was conducted with hnRNPK antibody. Western blotting was used to detect ubiquitinated-hnRNPK.

### *In vivo* tumor experiments

All animal studies in this study were conducted in accordance with the guidelines provided by the Animal Ethics Committee of Xi'an Jiaotong University. Four weeks female severe combined immunodeficiency (SCID) nude mice were obtained from the Laboratory Animal Center of Xi'an Jiaotong University and housed in a special pathogen-free animal facility. For orthotopic xenografts, 2×10^6^ cells diluted in 100 μl PBS were injected into the mammary fat pads of both sides. For lung metastasis model of tumor cells, 2×10^6^ cells suspended in 200μl PBS were injected via tail vein. At the end of the sixth week, mice were killed. The tumor incidence was examined by palpation and surgical incision of the mammary glands at the end of the experiment. Tumor weights were measured and tumor volumes were calculated by the formula (*a*×*b*^2^)/2, where *a* is the major tumor axis and *b* is the minor tumor axis. Lung tissues were collected for H-E (hematoxylin-eosin) staining. Relative number of lung metastases nodules was counted.

### Statistical analysis

All experiment were repeated at least three times *in vitro*, and all data were analyzed with the Graphpad Prism 8 (GraphPad Software, Inc., La Jolla, CA, USA). These results are expressed as the mean ± S.D. Comparisons between two groups were performed using Student's two-sided t-test. Pearson correlation analysis was used to detect the relationship between mRNA expression levels of PROX1 and EMT-related markers in TCGA database. The correlation between clinical characteristics and PROX1 expression levels in human breast cancer patients' samples were analyzed using the χ^2^ test. The preparation documents of the GSEA were all performed with R software (version 4.0.0; http://www.Rproject.org). P<0.05 was considered as statistically significant.

## Results

### PROX1 was up-regulated in breast cancer

**Figure 1:**
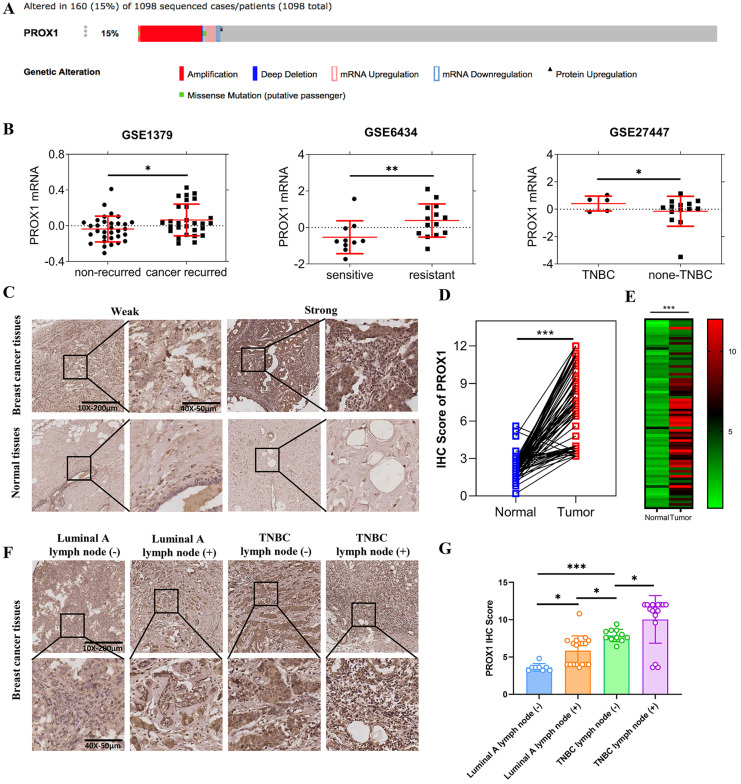
** The different expression of PROX1 in different subtypes of breast cancer. A.** The PROX1 alteration rate in 1098 sequenced patients by cBioportal. **B.** The scatter plots of PROX1 mRNA expression levels in different subgroups of breast cancer patients in GSE1379, GSE6434 and GSE27447 datasets from GEO databases respectively. **C.** Representative images of IHC staining results of PROX1 in breast cancer tissues and adjacent normal tissues. **D.** The IHC score analysis of PROX1 expression levels in paired breast cancer compared to matched normal tissues in our included patients' tissues. **E.** The heatmap analysis of PROX1 expression levels in paired breast cancer compared to matched normal tissues in our included patients' tissues. **F.** Representative images of IHC staining results of PROX1 in different subtypes of breast cancer tissues. G. Box plot showing the protein expression levels of PROX1 in different subtypes of breast cancer tissues. *P* values in B and G were calculated using unpaired two-tailed Student's *t* tests. *P* value in D and E was calculated using paired two-tailed Student's* t* tests. Abbreviation: TNBC: triple negative breast cancer; IHC: immunohistochemistry; GEO: Gene Expression Omnibus; *, *p*<0.05; **, *p*<0.01; ***, *p*<0.001.

In order to clarify the role of PROX1 in breast cancer, we searched for the mutation/loss/amplification of PROX1 in breast cancer from the tumor genome data analysis and open download platform cBioPortal for Cancer Genomics website (http://www.cbioportal.org/). Result showed that up to 15% of breast cancer patients were PROX1 amplified (Fig. 1A). In addition, the breast cancer RNA expression data were downloaded from Gene Expression Omnibus (GEO) database (http://www.ncbi.nlm.nih.gov/geo), including GSE1379, GSE6434 and GSE27447. GSE data analysis further showed that the expression of PROX1 was significantly increased in breast cancer patients with disease recurrence and docetaxel resistance. The expression of PROX1 in patients with triple-negative breast cancer was higher than that in patients with non-triple-negative breast cancer (Fig. 1B). Besides, immunohistochemical staining was used to analyze the expression of PROX1 in 74 pairs of breast cancer and normal tissues. The results showed that PROX1 expression was higher in cancer tissues compared with the matched adjacent normal tissues (Fig. 1C-E). Patients with lymph node metastasis and triple negative subtype acquired the highest expression of PROX1 (Fig. 1F). Statistical analysis also showed that the expression of PROX1 was the lowest in Luminal A subtype breast cancer patients without lymph node metastasis, while the expression was highest in triple-negative breast cancer patients with lymph node metastasis (Fig. 1G). Consistently, Table 1 confirmed that tumors with higher PROX1 expression was associate with larger tumor size, higher propensity for lymph node metastasis and triple negative subtype. Taken together, our data implied that higher PROX1 expression was correlated with malignancy and basal phenotype.

### PROX1 promoted the invasion and metastasis of breast cancer cells *in vitro*

**Figure 2:**
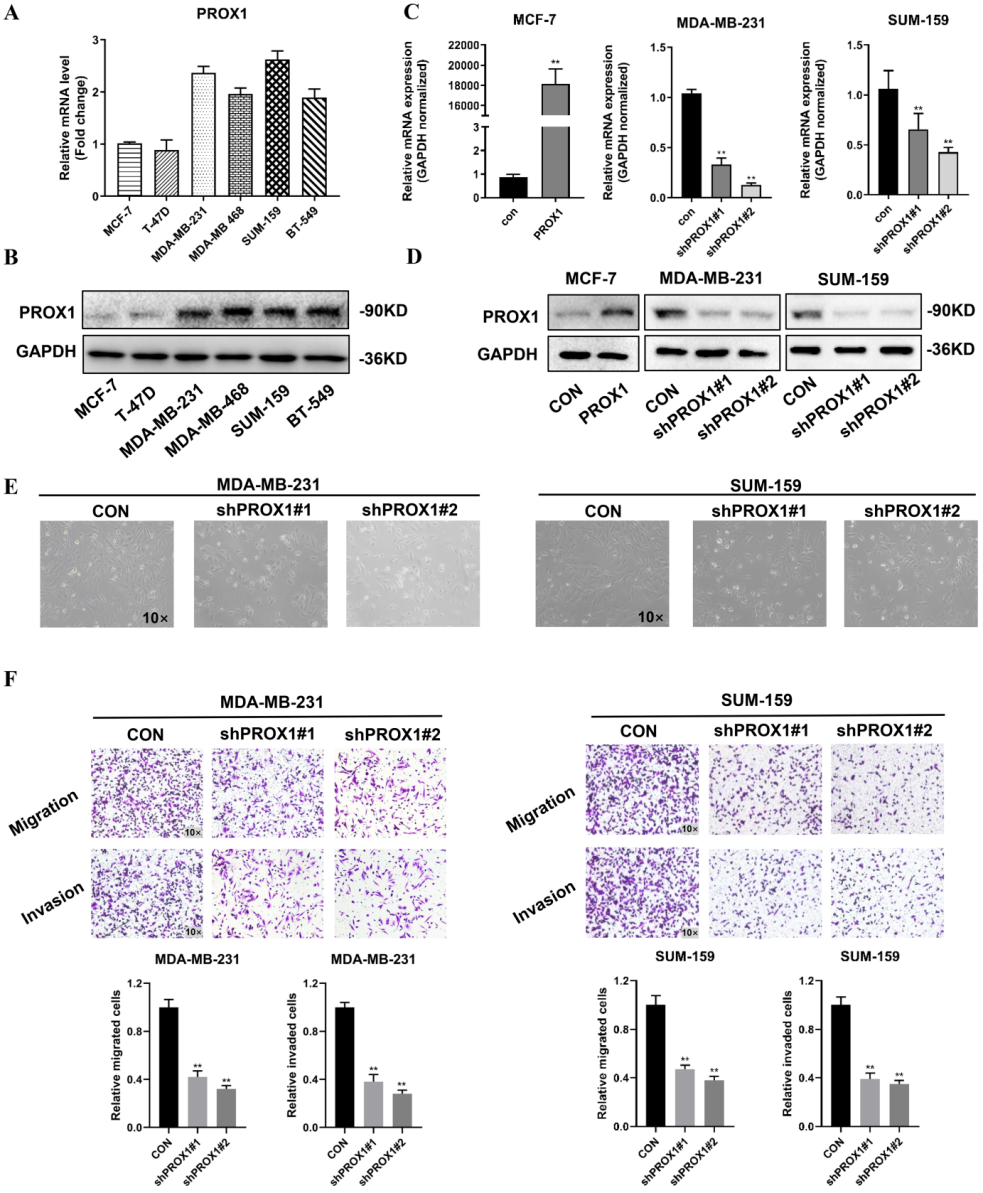
** PROX1 promotes the invasion and metastasis of breast cancer cells *in vitro*. A-B.** Expression of PROX1 mRNA (A) and protein (B) were examined in 6 kinds of cells by RT-qPCR and Western blotting. **C-D.** MCF-7 cells were transfected with lentiviral vectors encoding PROX1 (PROX1) or control vector (CON). MDA-MB-231 and SUM-159 cells were transfected with lentiviral vectors encoding PROX1 short hairpin RNA vector (shPROX1#1 or shPROX1#2) or control vector (CON). The expression of PROX1 was detected by RT-qPCR (C) and Western blotting (D). **E.** The brightfield images of MDA-MB-231 and SUM-159 cells' morphology after transfected with lentiviral vectors encoding PROX1 short hairpin RNA vector (shPROX1#1 or shPROX1#2) or control vector (CON). **F.** Cell migration and invasion assessed by transwell assay. The number of migrated or invaded cells were presented as the mean ± SD of three independent experiments. All the experiments were repeated three times independently with similar results. Data represent mean ± SD. Statistical significance was determined by two-tailed unpaired t-test. Abbreviation: *, *p*<0.05; **, *p*<0.01.

To elucidate role of PROX1 in breast cancer metastasis, we screened the mRNA and protein level of PROX1 in different cell lines. The expression of PROX1 were lower in T-47D and MCF-7 breast cancer cell lines, but higher in MDA-MB-231, MDA-MB-468, SUM-159 and BT-549 cell lines (Fig. 2A-B). MCF-7 was transfected with PROX1 plasmid and MDA-MB-231/SUM-159 was transfected with shPROX1 plasmid by lentivirus. The overexpression and knockdown efficiency of stable clones were detected by qRT-PCR and western blot (Fig. 2C-D). Morphological changes revealed that knockdown of PROX1 could lead to disorganization of the mesenchymal morphology of MDA-MB-231 and SUM-159 cell lines, leading to an epithelial morphology transition (Fig. 2E). We then performed transwell assays to explore the effect of PROX1 on cell migration and invasion. The overexpression of PROX1 gained stronger invasion and migration ability (Supplementary Fig. S1), while knockdown of PROX1 weakened the invasion and migration ability in breast cancer cells (Fig. 2F). Collectively, these data confirmed that PROX1 contributes breast cancer metastases *in vitro*.

### PROX1 promoted breast cancer progression by regulating EMT

**Figure 3:**
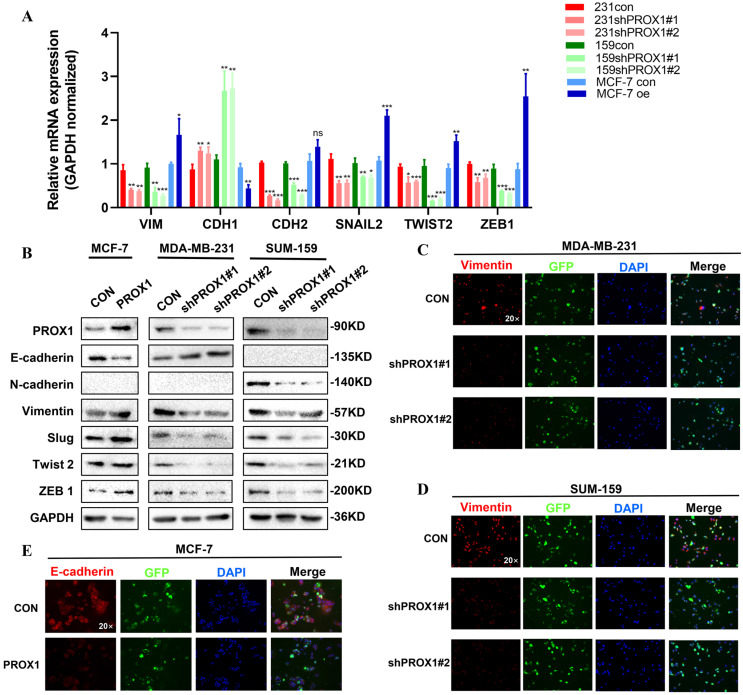
** PROX1 promotes breast cancer progression by regulating EMT. A-B.** RT-qPCR (A) and Western blotting (B) reveal the change of main epithelial markers and mesenchymal markers in of indicated PROX1 knockdown and overexpressed cell clones. **C-E.** Immunofluorescence staining reveal the change of epithelial markers and mesenchymal markers in of indicated PROX1 knockdown and overexpressed cell clones. All the experiments were repeated three times independently with similar results. Data represent mean ± SD.* P* values in A were calculated using unpaired two-tailed Student's *t* tests. Abbreviation: *, *p*<0.05; **, *p*<0.01; ***, *p*<0.001; ns, no significance.

Morphological changes indicated that PROX1 may contribute to EMT in breast cancer. Consistent with the morphological results, qRT-PCR result showed that knockdown of PROX1 could cause a significant decrease in the mRNA expression of VIM, CDH2, SNAIL2, TWIST2 and ZEB1 and vice versa (Fig. 3A). Besides, western blot also illustrated that ectopic overexpression of PROX1 decreased expression of E-cadherin, but increased the protein expression of Vimentin, Slug, Twist 2 and ZEB1. While knockdown of PROX1 down-regulated the protein expression of N-cadherin, Vimentin, Slug, Twist 2 and ZEB1 (Fig. 3B). Further, immunofluorescence staining results confirmed that the expression of Vimentin decreased in the PROX1 knock-down cells (Fig. 3C-D), while the expression of epithelial marker E-cadherin decreased in PROX1-overexpressed cells (Fig. 3E). Meanwhile, we also verified the correspondence between PROX1 and EMT related markers in the TCGA database. And the results were consistent with the cell experiment results (Supplementary Fig. S2). These data demonstrated that PROX1 was responsible for inducing EMT *in vitro*.

### PROX1 promoted EMT by facilitating nuclear translocation of β-catenin

**Figure 4:**
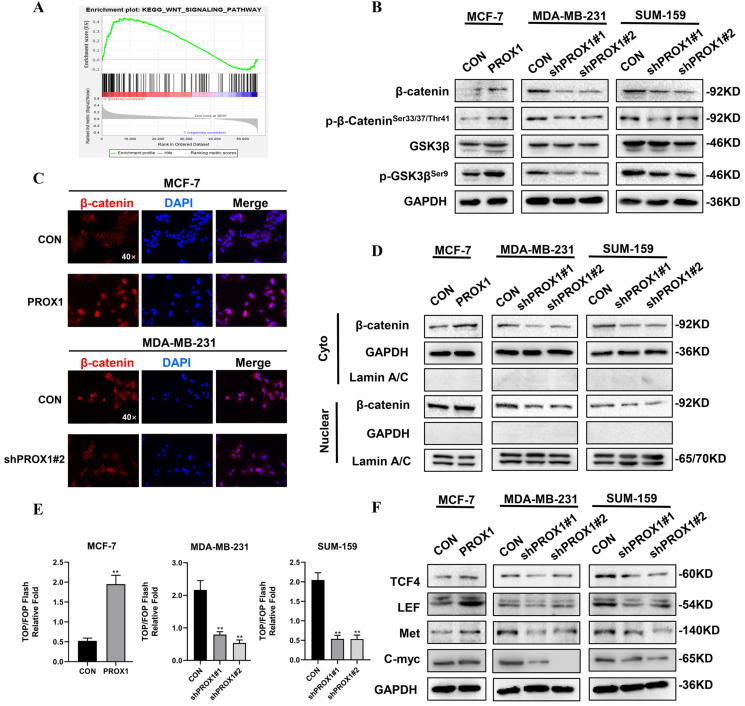
** PROX1 activates WNT signaling and promotes nuclear translocation of β-catenin. A.** The enrichment plot of WNT signaling pathway by GSEA analysis of significant pathway of PROX1 in breast cancer. **B.** Detection of β-catenin, p-β-catenin^Ser33/37/Thr41^, GSK3β and p-GSK3β^Ser9^ after PROX1 knockdown and overexpression by Western blotting. **C.** Localization of β-catenin after PROX1 knockdown and overexpression by immunofluorescence assays. **D.** Cytoplasmic and nuclear expression of β-catenin were determined by Western blotting. GAPDH was used as the loading control for the cytoplasmic protein. Lamin A/C was used as the loading control for nuclear protein. **E.** TOP/FOP luciferase reporter assays were detected in PROX1-knockdown and -overexpressing cells. Normalization was based on internal Renilla luciferase activity. The final reporter activity was measured as TOP/FOP ratio and was expressed as the mean ± SD of three independent experiments. **F.** Protein levels of WNT downstream targets were determined by Western blotting. All the experiments were repeated three times independently with similar results. Data represent mean ± SD.* P* values in E were calculated using unpaired two-tailed Student's *t* tests. *P* values in F were calculated using Pearson correlation analysis. Abbreviation: GSEA: Gene set enrichment analysis; **, *p*<0.01.

KEGG pathway analyzed by R software and GSEA 4.0.3 were used to guide us to find the mechanism of how PROX1 promoted EMT. The result showed that high PROX1 expression might promote WNT signaling pathway (Fig. 4A). To elucidate the role of PROX1 in WNT/β-catenin pathway, we detected the protein expression of these main regulators of WNT signaling. When knocking down PROX1 expression in MDA-MB-231 and SUM-159, both the total β-catenin level and phosphorylated β-catenin^Ser33/37/Thr41^ was decreased, while the phosphorylated of GSK3β^Ser9^ was decreased but the total GSK3β expression maintained. Consistently, the overexpression PROX1 in MCF-7 cell lines showed the consistent results (Fig. 4B). In addition to the protein expression levels of β-catenin, the subcellular localization of β-catenin was also crucial for measuring the activity of WNT pathway. Immunofluorescence staining showed that PROX1 could significantly promote the translocation of β-catenin into the nucleus in MCF-7, while the knockdown of PROX1 showed the reverse process in MDA-MB-231 (Fig. 4C). We then separated the cytoplasmic and nuclear protein extractions, results showed that both cytoplasmic and nuclear β-catenin levels were increased in PROX1 overexpressed MCF7 cells, while both cytoplasmic and nuclear β-catenin levels were decreased by PROX1 knockdown in MDA-MB-231 and SUM-159 cells (Fig. 4D). TOP/FOP luciferase reporter assays confirmed that β-catenin-driven transcription was activated in PROX1-overexpressed MCF-7 cells and vice versa (Fig. 4E). Moreover, WNT pathway controlled the transcription of many important genes including TCF4, LEF, Met and C-myc. Results showed that the protein levels of these downstream target genes were upregulated in PROX1 overexpressed MCF-7 cells and downregulated in PROX1 knockdown MDA-MB-231 and SUM-159 cells (Fig. 4F). Meanwhile, both the Wnt/β-catenin signaling-specific inhibitor XAV-939 and Wnt/β-catenin activator SKL2001 were utilized to analyze the Wnt/β-catenin signaling in MCF-7 and MDA-MB-231 cells respectively. Results showed that the XAV-939 could significantly abolish the promotion of migration in PROX1-overexpressing MCF-7 cells, while SKL2001 could obviously retrieve the inhibition of migration in PROX1-knockdown MDA-MB-231 cells. Meanwhile, we also observed that changes of E-cadherin reasoned by PROX1 interfering or upregulation can be recovered by SKL2001 or XAV939, respectively (Supplementary Fig. S3). Collectively, it highly demonstrated that PROX1 possibly promoted EMT process through Wnt/β-catenin pathway and the observed migration-metastasis phenotype was mainly dependent on WNT/β-catenin.

### Knockdown of PROX1 inhibited tumor growth and metastasis of breast cancer *in vivo*

**Figure 5:**
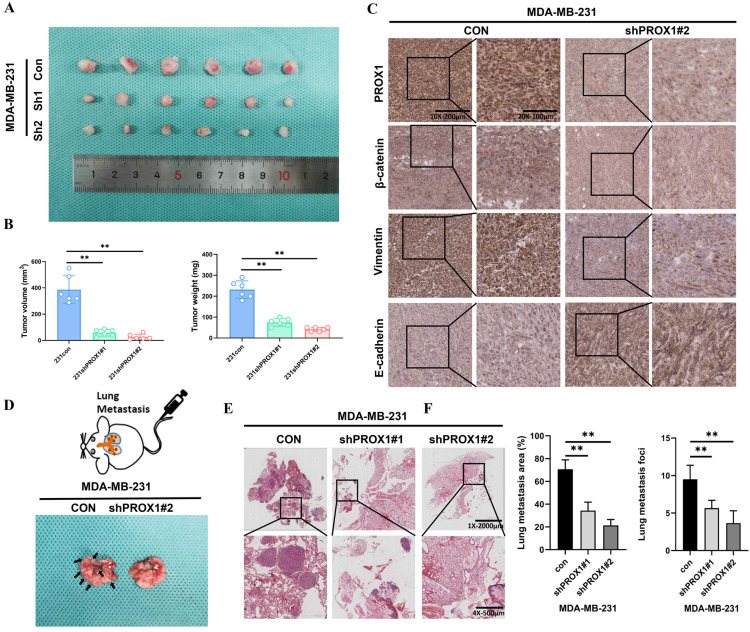
** Knockdown of PROX1 inhibits tumor growth and metastasis of breast cancer *in vivo*. A.** PROX1 knockdown inhibited subcutaneous tumor formation in a nude mouse model. **B.** Tumor volume and weight analysis of subcutaneous tumors in nude mouse models. **C.** Representative IHC staining results of tumor samples. **D-E.** Representative visualized lung metastasis models (D) and HE staining of lung metastasis specimen (E) (arrows: metastatic foci). **F.** Number of metastatic foci and lung metastasis area in control and PROX1 knockdown MDA-MB-231 cells. Statistical significance was determined by two-tailed unpaired *t*-test. Abbreviation: **, *p*<0.01.

Nude mice bearing MDA-MB-231 xenograft tumors were used to investigate whether PROX1 could still promotes tumor proliferation and metastasis *in vivo*. MDA-MB-231/Con, MDA-MB-231/shPROX1#1 and MDA-MB-231/shPROX1#2 cells were subcutaneously inoculated into the nude mouse models. Mice in the shPROX1 groups developed smaller tumors than those in control group (Fig. 5A). Tumor volumes and tumor weights were also obviously reduced in mice inoculated with PROX1 knockdown cells (Fig. 5B). IHC analyses showed that the knockdown of PROX1 could significantly inhibit the expression of β-catenin and vimentin, but promoted the expression of E-cadherin in transplanted tumor tissues (Fig. 5C). To determine metastasis activity of PROX1 *in vivo*, MDA-MB-231/Con and MDA-MB-231/shPROX1#1&#2 cells were injected into the caudal vein of balb/c nude mice to construct lung metastasis mode. Results showed that the number and volume of lung metastases were significantly reduced in PROX1 knockdown group (Fig. 5D-F). These data indicated that knockdown of PROX1 could inhibits tumorigenesis and distant metastasis *in vivo*.

### PROX1 interacted with hnRNPK and upregulated its expression by preventing hnRNPK ubiquitination

**Figure 6:**
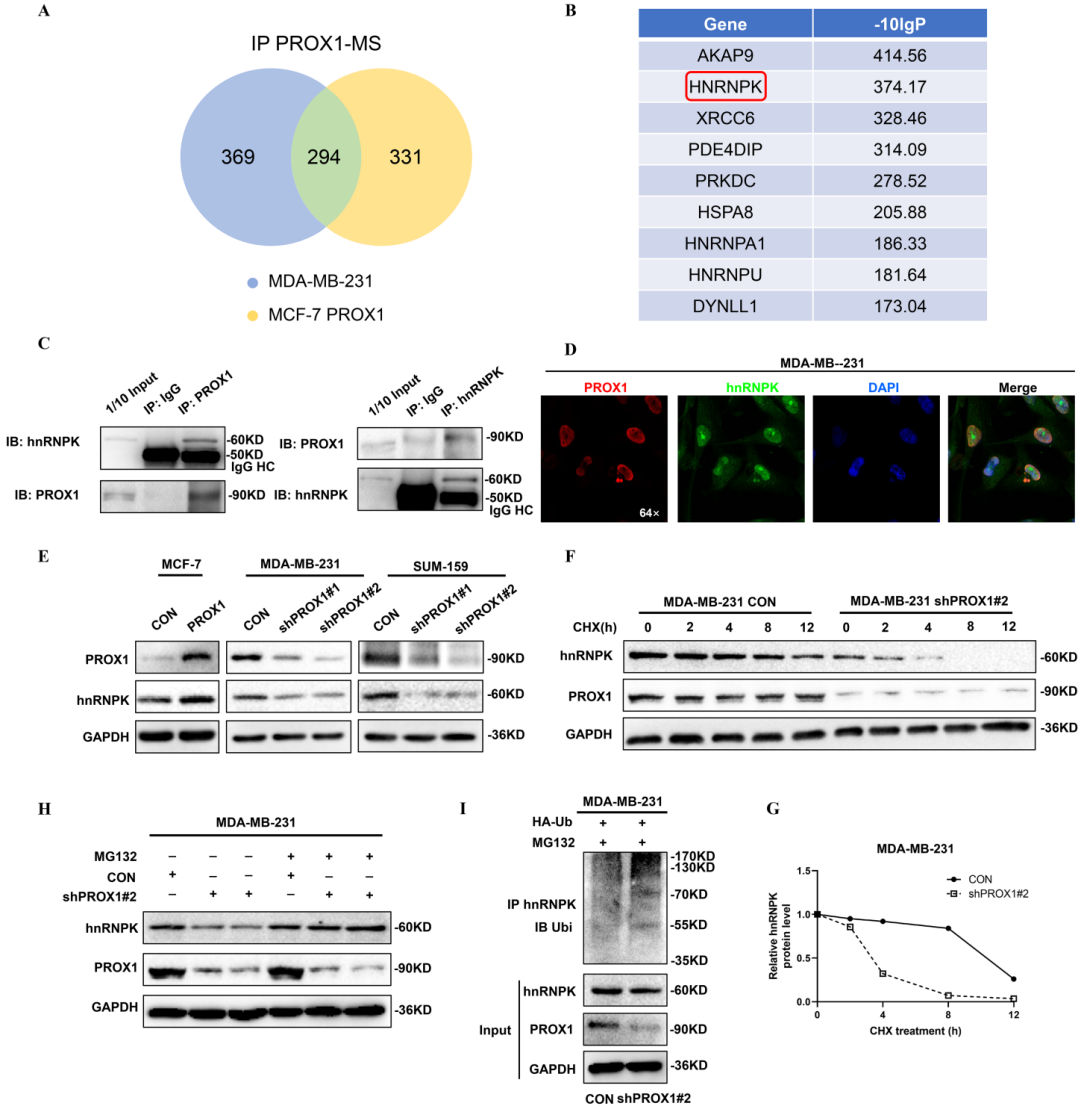
** PROX1 interacts with hnRNPK and upregulates its expression by preventing hnRNPK ubiquitination. A.** Venn diagram showing the common PROX1 binding proteins in MASS analyses of Co-IP samples (IP PROX1 in MDA-MB-231 and IP PROX1 in PROX1 overexpressed MCF-7 cells). **B.** The top lists PROX1 binding proteins in MASS analyses. **C.** Co-IP analysis of interaction between PROX1 and hnRNPK in MDA-MB-231 cells. **D.** Co-localization of PROX1 (red) and hnRNPK (green) was indicated by immunofluorescence confocal microscopy. DAPI was used for nuclear staining (blue). **E.** Expression of hnRNPK in PROX1 -knockdown and -overexpressing cells were determined by Western blotting. **F-G.** Cycloheximide (100 ug/ml) contact MDA-MB-231 cells with gradient time. The protein expression was examined by Western blotting (F). Quantification of hnRNPK protein levels was determined using Image J software normalized to GAPDH (G). **H.** MG132 (5 μM) contact MDA-MB-231 cells with 8h. The protein expression was examined by Western Blotting. **I.** The ubiquitination of hnRNPK was examined when PROX1 was knocked down in MDA-MB-231 cells. Ubiquitin-HA was transfected into MDA-MB-231 CON and MDA-MB-231 shPROX1#2 cells. Ubiquitination hnRNPK was tested by IP of hnRNPK and analyzed by anti-ubiquitin antibody with Western blotting. All the experiments were repeated three times independently with similar results.

We further explored the mechanism of how PROX1 activate WNT signaling. Co-immunoprecipitation followed by mass spectrometry were used to identify the PROX1 interacting proteins. Venn diagram showing the common PROX1 binding proteins in MASS analyses of Co-IP samples in both MDA-MB-231 and PROX1 overexpressed MCF-7 cells (Fig. 6A). Heterogeneous ribonucleoprotein K (hnRNPK) was identified by mass spectrometry from the top list and confirmed by western blot after Co-IP (Fig. 6B-C, Supplementary Fig. S5A). Physical interaction evidence between PROX1 and hnRNPK provided by AI calculation were shown in Supplementary Fig. S5B. Previous study detected that hnRNPK could interact with β-catenin to alter downstream gene expression and shuttling between the nucleus and cytoplasm 14. Our GSEA data suggested that PROX1 was positively correlated with WNT pathway activation while β-catenin was crucial in WNT signal transduction. Therefore, we hypothesized that PROX1 could modulate WNT/β-catenin signaling through interacting with hnRNPK. We investigated the mRNA expression of hnRNPK in breast cancer cell lines with altered PROX1 expression. No fold change in mRNA level of hnRNPK suggested that the regulation was post-transcriptional (Supplementary Fig. S4). Immunofluorescence experiment was used to detect the localization of both PROX1 and hnRNPK in MDA-MB-231 cells. Results showed that PROX1 (red) and hnRNPK (green) formed an overlapping staining signal (yellow) in nucleus (Fig. 6D). In addition, the protein levels of hnRNPK were assessed by western blot. Results showed that hnRNPK levels were increased in PROX1 overexpressed in MCF-7 cells, while hnRNPK expression was reduced in PROX1 knockdown cells of MDA-MB-231 and SUM-159 (Fig. 6E).

To further elucidate how PROX1 regulated protein expression of hnRNPK, we investigated the half-lives of hnRNPK influenced by PROX1 in MDA-MB-231 cells. The results showed that the half-life of hnRNPK was reduced without PROX1, which indicating PROX1 maintained the stability of hnRNPK (Fig. 6F-G). Treatment with MG132 could also abolish the decrease of hnRNPK in PROX1 depleted MDA-MB-231 cells (Fig. 6H). As for the mechanism of PROX1 regulating hnRNPK protein degradation, immunoprecipitation was performed on MDA-MB-231 cell sublines and the data showed that ubiquitin of hnRNPK was significantly increased without PROX1 (Fig. 6I). Therefore, the data confirmed that PROX1 regulated the degradation of hnRNPK by ubiquitin pathway.

### hnRNPK facilitated PROX1 induced WNT signaling activation in breast cancer cells

**Figure 7:**
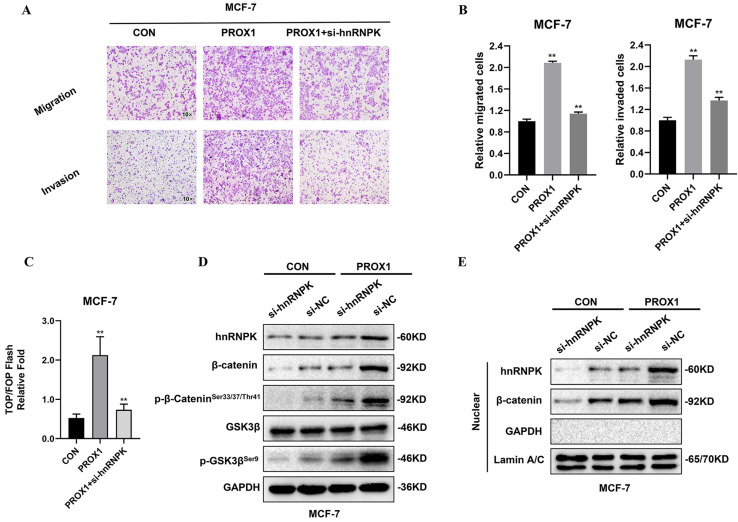
** hnRNPK facilitate PROX1 induced WNT signaling activation in breast cancer cells. A-B.** Cell migration and invasion assessed by transwell assay. The number of migrated or invaded cells were presented as the mean ± SD of three independent experiments. **C.** TOP/FOP luciferase reporter assays were detected in PROX1 overexpressing cells with or without transfected si-hnRNPK. Normalization was based on internal Renilla luciferase activity. The final reporter activity was measured as TOP/FOP ratio and was expressed as the mean ± SD of three independent experiments. **D.** The protein levels of hnRNPK, β-catenin, p-β-catenin^Ser33/37/Thr41^, GSK3β and p-GSK3β^Ser9^ were determined by Western blotting after inhibiting the expression of hnRNPK in PROX1 overexpressed MCF-7 cells. **E.** The nuclear protein levels of hnRNPK and β-catenin were examined by Western blotting after we increased the expression of PROX1 but inhibited the expression of hnRNPK. GAPDH was used as the loading control for the cytoplasmic protein. Lamin A/C was used as the loading control for nuclear protein. All the experiments were repeated three times independently with similar results. Data represent mean ± SD.* P* values in B and C were calculated using unpaired two-tailed Student's *t* tests. Abbreviation: **, *p*<0.01.

To further identify whether hnRNPK played the crucial role in WNT activation by PROX1, hnRNPK was knocked down by siRNA. TOP/FOP luciferase reporter assays and transwell assays were performed to detect the cell migration and invasion as well as transcriptional activity of β-catenin. Results showed that the knockdown of hnRNPK could abolish PROX1 overexpression induced β-catenin activation in MCF-7 cells (Fig. 7A-C). Moreover, the depletion of hnRNPK could also abolished the up-regulation of β-catenin, the phosphorylation of β-catenin in Ser33/37/Thr41 and the phosphorylation of GSK3β in Ser9 in MCF-7 cell lines with overexpressed PROX1 (Fig. 7D). Nuclear localization of β-catenin was subsequently decreased by the knockdown of hnRNPK in PROX1 overexpressed cells (Fig. 7E). These data suggested that PROX1 promoted β-catenin nuclear accumulation in hnRNPK-dependent manner.

## Discussion

PROX1 is a member of the homeobox transcription factor family and is essential for organ development during embryogenesis. The Drosophila homolog called prospero has been shown to inhibit tumor progression by controlling the asymmetric cell division of neuroblasts 15. At the same time, studies have found that the alteration of PROX1 are related to the malignant biological behavior of different cancers, and also involved in the occurrence and progression of cancer. The function of this gene is still controversial in different cancer types. Previous studies had shown that altered PROX1 expression in advanced malignancies is a main regulator for tumor metastases. Chen's group demonstrated that exosomal LNMAT2 could epigenetically upregulate PROX1 expression and ultimately result in lymph-angiogenesis and lymphatic metastasis 16. Yokobori revealed that nuclear PROX1 was positively correlated with hypoxia-inducible factor 1α (HIF1α) expression and cancer progression in esophageal squamous cell carcinoma 11. Our previous study also confirmed that DAB2IP could promote EMT and metastasis in prostate cancer by inhibiting proteasome degradation of HIF1α through targeting PROX1 transcription^17^.

In this study, we found that PROX1 was frequently up-regulated in breast cancer patients and acted like oncogene by inducing EMT *in vitro* and *in vivo*. GSEA analysis based on TCGA database showed that high PROX1 expression might promote WNT signaling pathway. WNT signaling has become a basic pathway to control growth in not only embryonic development, but also animal evolution and cancer 18. We speculated that PROX1 may affect the WNT pathway in many different ways and we detected some important markers about Wnt pathway including total and phosphor GSK3β. We found that phosphorylation of GSK3β and β-catenin was decreased by PROX1 knockdown, in contrast, the phosphorylation levels of GSK3β and β-catenin was increased by PROX1 overexpression. The change of phosphor GSK3β reminded us of the crosstalk between pathways might indirectly affect WNT signaling in upstream of pathway. We chose to focus on β-catenin because it's the downstream of the pathway which has a more direct effect. WNT activation binds to different types of receptors, activates a variety of signal transduction pathways, generates a series of integrated EMT-related signals and induces cell proliferation and regular arrangement. The abnormal activation of the WNT/β-catenin pathway is widespread in different kinds of human cancers. We found that PROX1 promoted cancer metastasis by activating WNT signaling in maintaining β-catenin stability and promoting nuclear translocation of β-catenin. Since we didn't see any changes in β-catenin mRNA, we considered that regulation was done in protein level.

**Figure 8:**
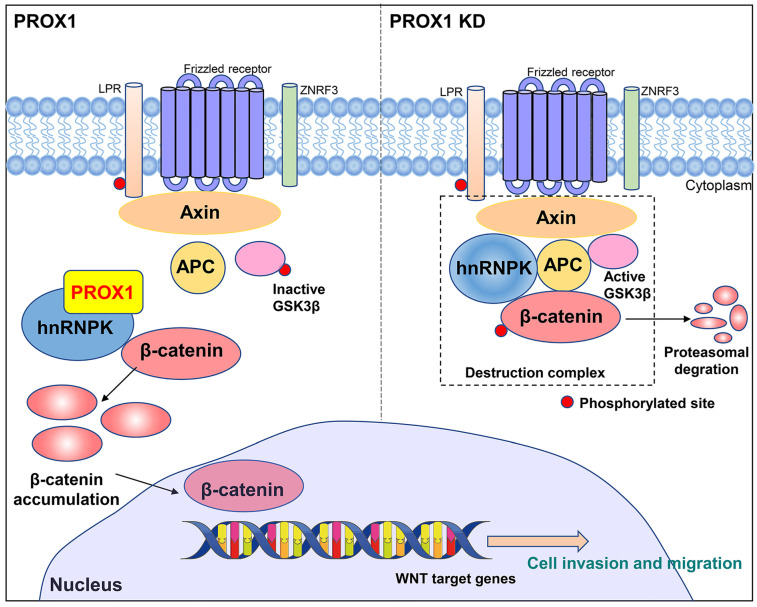
A schematic model of the role of PROX1 in breast cancer.

Co-immunoprecipitation and mass spectrometry were performed to identify the PROX1 interacting proteins. According to our IP-MASS data, PROX1 did not directly interact with any classical protein in WNT pathway. However, several members in heterogeneous ribonucleoprotein family from the top list showed physically binding with PROX1. Among them, hnRNPK caught our attention and confirmed the binding with PROX1 by western blot after CO-IP. hnRNPK has been proved and reported to interact with β-catenin to alter downstream gene expression and shuttle it between the nucleus and cytoplasm, in addition, it can also interact with AXIN, GSK3β and TCF4 to form a complex by coimmunoprecipitation. The interaction between hnRNPK and those markers above suggested that hnRNPK might protect β-catenin from degradation 19. hnRNPK is a regulatory protein in the heterogeneous nuclear ribonucleoprotein family. hnRNPs are widely expressed by a variety of human cells, and are located in the nucleus, cytoplasm, mitochondria and cell membrane. These proteins form a ribonucleoprotein complex, which is involved in many cellular processes, including gene expression, cell signal transduction, DNA repair and telomere synthesis 20. hnRNPK is especially central to many biological pathways including gene expression regulation and cell signal transduction 21. Genetic knockout of hnRNPK in mice resulted in embryonically lethal, suggestive of its critical role in neonatal development and survival 22. In addition, hnRNPK is involved in multiple types of cancers where it is often aberrantly expressed compared with the normal tissue cells, and also essential for cancer metastasis by inducing cell movement, angiogenesis and extracellular matrix modifying genes 23-26. In order to elucidate whether hnRNPK was crucial for PROX1 to activate WNT pathway, we manipulated hnRNPK level and used TOP/FOP luciferase assay to monitor WNT/β-catenin signaling activity. Results also showed that the level of β-catenin-driven transcription was reversed after knockdown hnRNPK in PROX1 overexpressed MCF-7 cells. We further confirmed that the knockdown of PROX1 in MDA-MB-231 cells could increase the ubiquitination of hnRNPK. Taken together, these data showed that PROX1 maintained the stability of hnRNPK which subjects to activate WNT signaling by facilitating transporting β-catenin into nucleus (Fig. 8).

## Conclusion

In conclusion, PROX1 exerts its pro-metastasis role in breast cancer by interacting with hnRNPK to activate WNT/β-catenin signaling to promote the invasion and metastasis of breast cancer. Thus, PROX1 could act as an important diagnostic marker and also significant therapeutic target for breast cancer patients.

## Supplementary Material

Supplementary figures and tables.

## Figures and Tables

**Table 1:** Association of PROX1 level with clinicopathological parameters of patients with breast cancer

Characteristics	Total	PROX1		P value
		Low	High	
**Age (y)**				
≤ 60	43	17	26	0.218
> 60	31	8	23	
**Tumor size**				
≤ 2 cm	13	8	5	0.045*^, a^
> 2 cm	61	17	44	
**T**				
T1-T2	63	21	42	1.000^ a^
T3-T4	11	4	7	
**N**				
N0	24	12	12	0.041*
N1-N3	50	13	37	
**Pathological grade**				
I-II	46	17	29	0.460
III	28	8	20	
**AJCC stage**				
IA-IIB	40	16	24	0.220
IIIA- IIIC	34	9	25	
**ER status**				
negative	54	12	42	0.001*
positive	20	13	7	
**PR status**				
negative	44	8	36	0.001*
positive	30	17	13	
**Her2 status**				
negative	55	20	35	0.425
positive	19	5	14	
**TNBC**				
no	47	22	25	0.002*
yes	27	3	24	

*Represents statistically significant; ^a^ represents the statistical method of successive corrections.
