# Supplementary material for: PROX1 promotes breast cancer invasion and metastasis through WNT/β-catenin pathway via interacting with hnRNPK
Source: Int J Biol Sci. 2022 Feb 28;18(5):2032–46. doi: 10.7150/ijbs.68960 (PMC8935233; doi:10.7150/ijbs.68960)
Supplement: Supplementary file 1 — Supplementary figures and tables. [file ijbsv18p2032s1.pdf]

Supplementary Figure 1 Cell migration and invasion of MCF-7 CON and PROX1 overexpressed were assessed by transwell assay. The number of migrated or invaded cells were presented as the mean ± SD of three independent experiments.
Statistical significance was determined by two-tailed unpaired *t*-test.
Abbreviation: **, p<0.01.

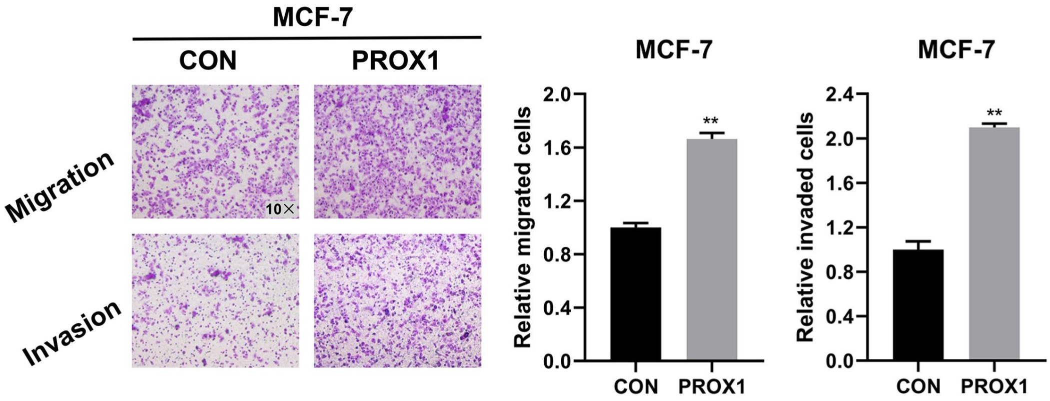

Supplementary Figure 2 Scatter plots of the relationship between mRNA expression levels of PROX1 and EMT-related markers in TCGA database. "n" represents the number of included patients.
P values were calculated using Pearson correlation analysis.
Abbreviation: TCGA: the Cancer Genome Atlas.

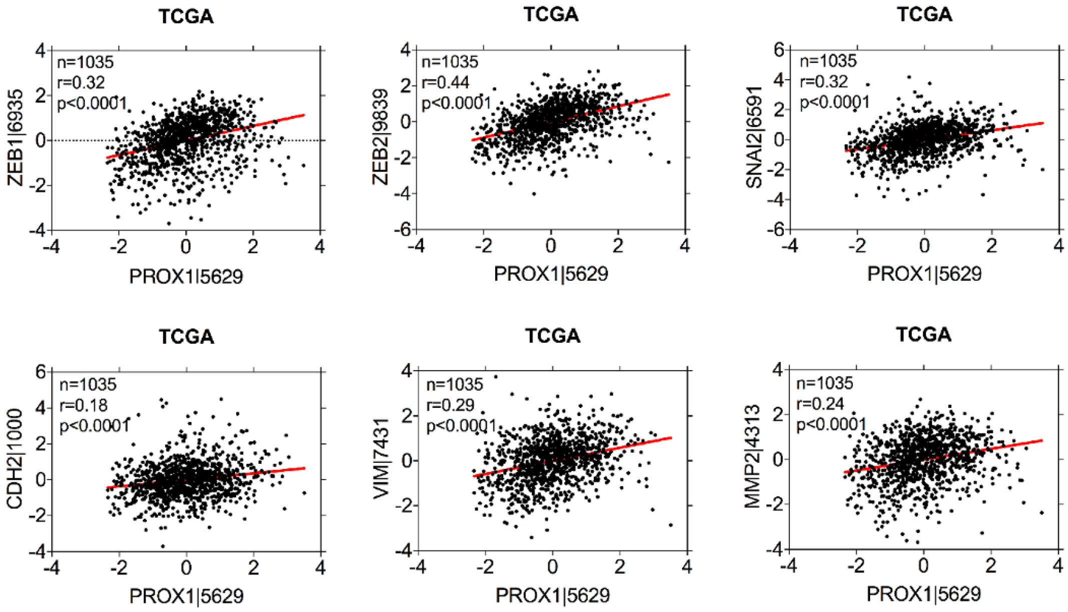

Supplementary Figure 3 PROX1 promotes EMT through Wnt/β-catenin signaling pathway. A-B. Rescue transwell migration assay of PROX1-knockdown and -overexpressing cells which were pretreated with SKL2001 or XAV939 (either 30μM SKL2001 or 20μM XAV939 for 24h). The number of migrated cells were presented as the mean ± SD of three independent experiments. C. The protein level of E-cadherin in PROX1-knockdown and -overexpressing cells (either 30μM SKL2001 or 20μM XAV939 for 24h) were assessed by Western blotting.
All the experiments were repeated three times independently with similar results. Data represent mean ± SD. *P* values in B were calculated using unpaired two-tailed Student's *t* tests.
Abbreviation: **, *p*<0.01.

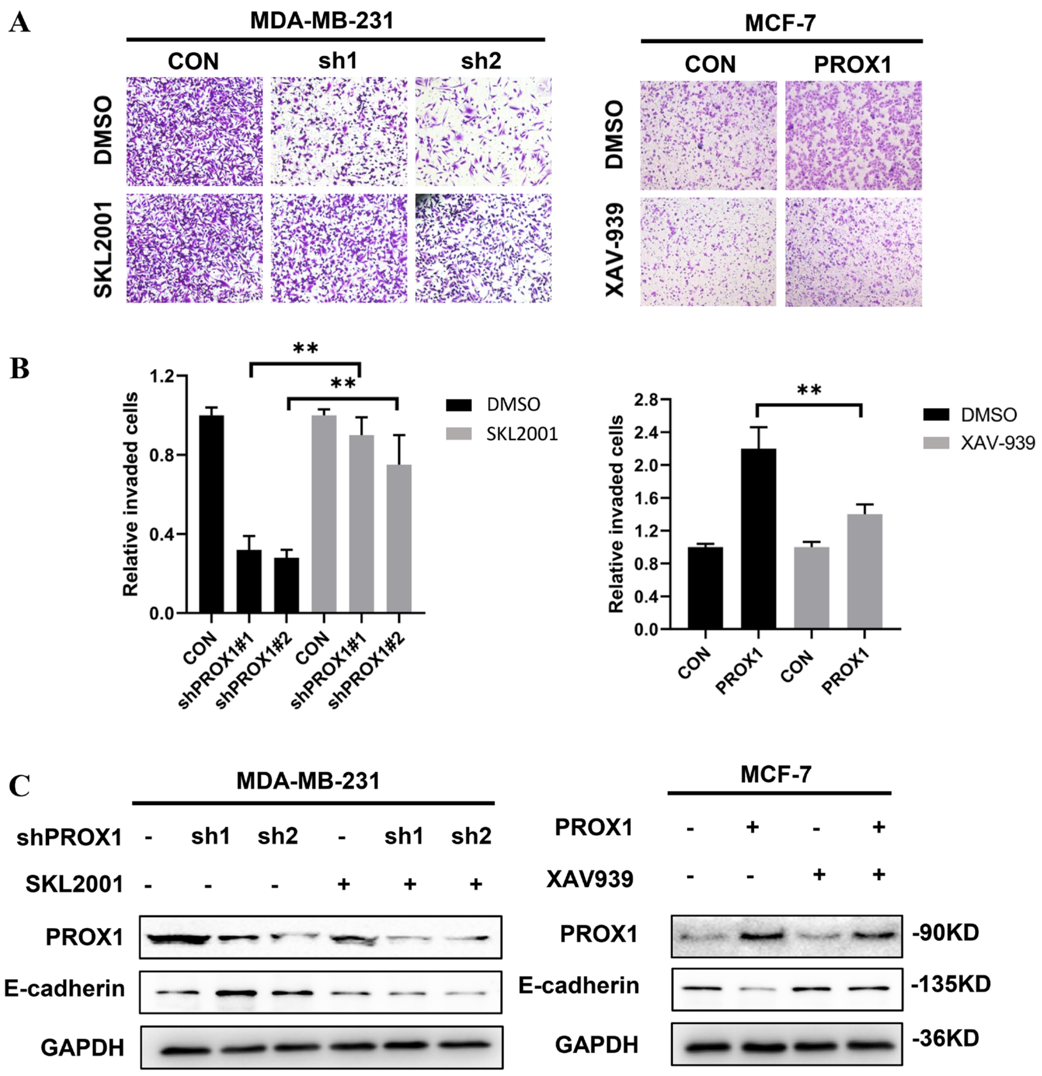

Supplementary Figure 4 The mRNA expression of hnRNPK in PROX1-knockdown and -overexpressing cells were detected by RT-qPCR.

The experiments were repeated three times independently with similar results. Data represent mean ± SD. *P* values were calculated using unpaired two-tailed Student's *t* tests.

Abbreviation: ns: no significance.

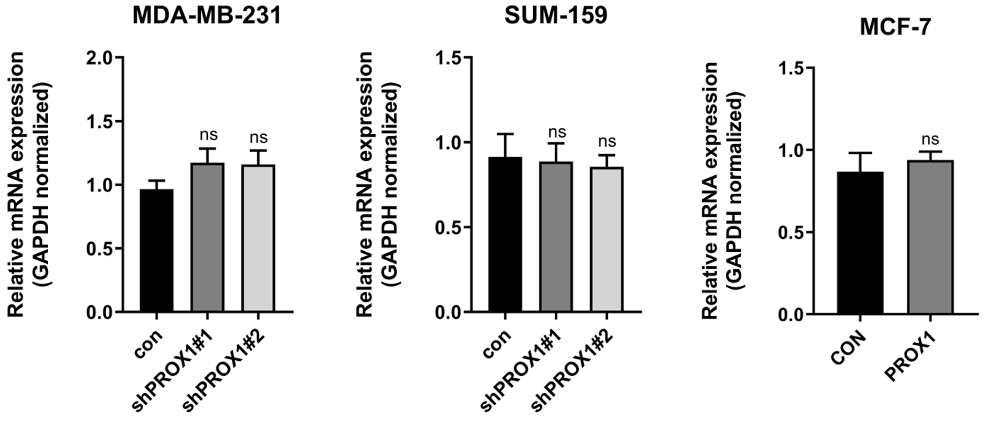

Supplementary Figure 5 The interaction between PROX1 and hnRNPK. A. Co-IP analysis of interaction between PROX1 and hnRNPK in MCF-7 PROX1 cells. B. Physical interaction evidence between PROX1 and hnRNPK provided by AI calculation.

ΔiG P-value indicates the P-value of the observed solvation free energy gain. The P-value measures the probability of getting a lower than observed ΔiG, when the interface atoms are picked randomly from the protein surface, such as to amount to the observed interface area. The P-value is a measure of interface specificity, showing how surprising, in energy terms, the interface is. ΔiG indicates the solvation free energy gain upon formation of the interface, in kcal/M. The value is calculated as difference in total solvation energies of isolated and interfacing structures. Negative ΔiG corresponds to hydrophobic interfaces, or positive protein affinity.

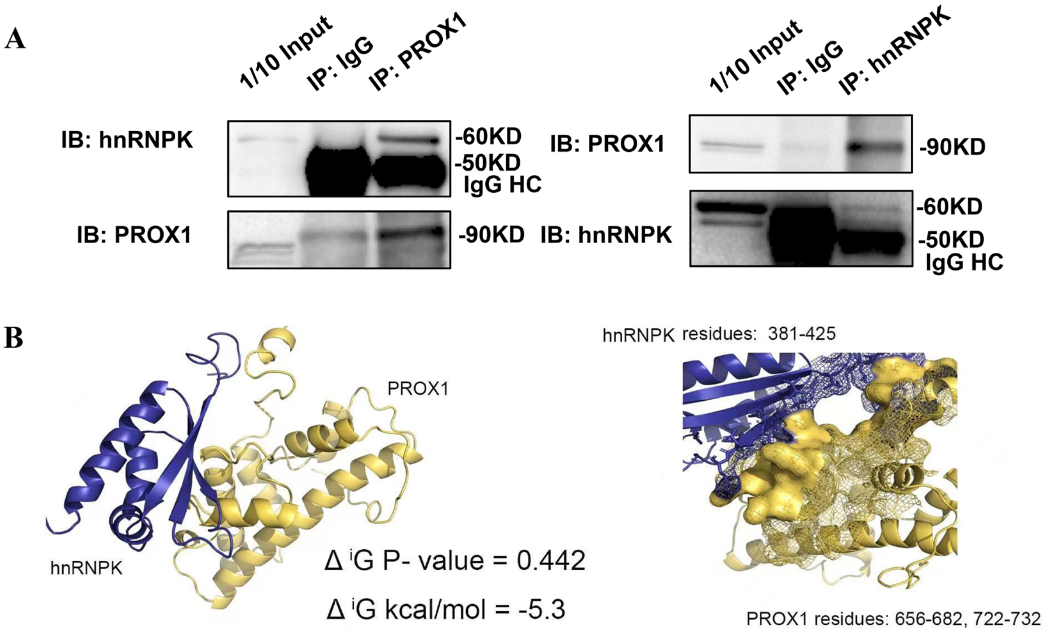
A
1/10 Input
IP: IgG
IP: PROX1
IB: hnRNPK
-60KD
-50KD
IgG HC
IB: PROX1
-90KD
1/10 Input
IP: IgG
IP: hnRNPK
IB: PROX1
-90KD
IB: hnRNPK
-60KD
-50KD
IgG HC
B
PROX1
hnRNPK
Δ iG P- value = 0.442
Δ iG kcal/mol = -5.3
hnRNPK residues: 381-425
PROX1 residues: 656-682, 722-732

Supplementary Table 1. The antibodies list used in this study.

Name	Source	Catalog number
PROX1	Proteintech	11067-2-AP
GAPDH	Proteintech	HRP-60004
E-cadherin	Cell Signaling Technology	#3195
N-cadherin	Cell Signaling Technology	#13116
Vimentin	Cell Signaling Technology	#5741
Slug	Cell Signaling Technology	#9585
Twist2	Proteintech	11752-1-AP
ZEB1	Cell Signaling Technology	#70512
β-catenin	Cell Signaling Technology	#8480
p-β-catenin$^{Ser33/37/Thr41}$	Cell Signaling Technology	#9561
GSK3β	Cell Signaling Technology	#12456
p-GSK3β^{Ser9}	Cell Signaling Technology	#9322
Lamin A/C	Proteintech	10298-1-AP
TCF4	Santa Cruz	sc-166699
LEF	Santa Cruz	sc-374412
Met	Cell Signaling Technology	#8198
C-myc	Proteintech	10828-1-AP
hnRNPK	Abcam	ab52600
Ubiquitin	Santa Cruz	sc-8017

Supplementary Table 2. The sequences of shRNA, siRNA and primers for real-time PCR assays.

Gene	Sequence
shPROX1#1	GAAGTTGCTCAGATCACATTA
shPROX1#2	TTTCCAGGAGCAACCATAATT
sihnRNPK Sense	GCAUAAAGAUCAUCCUUGA
sihnRNPK Antisense	UCAAGGAUGAUCUUUAUGC
GAPDH-F	GGAGCGAGATCCCTCCAAAAT
GAPDH-R	GGCTGTTGTCATACTTCTCATGG
PROX1-F	AAAGTCAAATGTACTCCGCAAGC
PROX1-R	CTGGGAAATTATGGTTGCTCCT
hnRNPK-F	GCAGGAGGAATTATTGGGGTC
hnRNPK-R	TGCACTCTACAACCCTATCGG
VIM-F	AGTCCACTGAGTACCGGAGAC
VIM-R	CATTTCACGCATCTGGCGTTC
CDH1-F	CGAGAGCTACACGTTCACGG
CDH1-R	GGGTGTCGAGGGAAAAATAGG
CDH2-F	TGCGGTACAGTGTAACTGGG
CDH2-R	GAAACCGGGCTATCTGCTCG
SNAIL2-F	TGTGACAAGGAATATGTGAGCC
SNAIL2-R	TGAGCCCTCAGATTTGACCTG
TWIST2-F	CGCAAGTGGAATTGGGATGC
TWIST2-R	CGATGTCACTGCTGTCCCTT
ZEB1-F	TTCAAACCCATAGTGGTTGCT
ZEB1-R	TGGGAGATACCAAACCAACTG
